# Landmark-Based Scale Estimation and Correction of Visual Inertial Odometry for VTOL UAVs in a GPS-Denied Environment

**DOI:** 10.3390/s22249654

**Published:** 2022-12-09

**Authors:** Jyun-Cheng Lee, Chih-Chun Chen, Chang-Te Shen, Ying-Chih Lai

**Affiliations:** 1 Department of Aeronautics and Astronautics, College of Engineering, National Cheng Kung University, Tainan 701, Taiwan; 2 Institute of Civil Aviation, College of Engineering, National Cheng Kung University, Tainan 701, Taiwan

**Keywords:** data fusion, non-GPS navigation, scale correction, visual inertial odometry, artificial landmark, extended Kalman filter

## Abstract

With the rapid development of technology, unmanned aerial vehicles (UAVs) have become more popular and are applied in many areas. However, there are some environments where the Global Positioning System (GPS) is unavailable or has the problem of GPS signal outages, such as indoor and bridge inspections. Visual inertial odometry (VIO) is a popular research solution for non-GPS navigation. However, VIO has problems of scale errors and long-term drift. This study proposes a method to correct the position errors of VIO without the help of GPS information for vertical takeoff and landing (VTOL) UAVs. In the initial process, artificial landmarks are utilized to improve the positioning results of VIO by the known landmark information. The position of the UAV is estimated by VIO. Then, the accurate position is estimated by the extended Kalman filter (EKF) with the known landmark, which is used to obtain the scale correction using the least squares method. The Inertial Measurement Unit (IMU) data are used for integration in the time-update process. The EKF can be updated with two measurements. One is the visual odometry (VO) estimated directly by a landmark. The other is the VIO with scale correction. When the landmark is detected during takeoff phase, or the UAV is returning to the takeoff location during landing phase, the trajectory estimated by the landmark is used to update the scale correction. At the beginning of the experiments, preliminary verification was conducted on the ground. A self-developed UAV equipped with a visual–inertial sensor to collect data and a high-precision real time kinematic (RTK) to verify trajectory are applied to flight tests. The experimental results show that the method proposed in this research effectively solves the problems of scale and the long-term drift of VIO.

## 1. Introduction

UAVs are unmanned, thereby reducing casualties caused by accidents, and they have many applications, including 3D mapping, entertainment, object detection, aerial photography for various purposes, logistics, military, and agriculture. However, there are some environments where the GPS or Global Navigation Satellite System (GNSS) signals are unavailable or have the problem of signal outages, such as indoor and bridge inspections. Therefore, finding an alternative localization method for UAVs in GPS-denied environments is necessary. Many non-GPS navigation methods have been proposed in these years [1,2]. Most of them can be used in indoor and outdoor environments, but their performance strongly depends on the environment conditions, coverage area, and sensor characteristics. Sensor selection is a crucial step for mission execution and is directly affected to task performance. UAVs with light detection and ranging (LiDAR) sensors can yield accurate measurements [3]. However, LiDAR sensors are not suitable for small or micro UAVs because they are heavier and more expensive than other sensors.

On the other hand, radio has the advantages of low price and lightweight. However, researchers have used radio frequency (RF) in specified areas or limited indoor environments because of its requirement for network infrastructure and fingerprint, trilateration, or triangulation methods [4]. A camera is also a popular device for UAV navigation in a GPS-denied environment due to its low cost, small size, and provision of rich information [4,5]. In contrast to RF navigation, visual simultaneous localization and mapping (vSLAM) [5] and VO do not need to set up the infrastructure and build the coverage map in advance. A real-time visual SLAM is proposed by Davison [6]. In this study, the algorithm adopts only a monocular camera and multiple pictures to rebuild the map and estimate localization and the attitude of the camera. Some popular SLAM methods including ORB-SLAM [7,8,9], LSD-SLAM [10], and DSO-SLAM [11] have been developed in these years. Most of them have high accurate results in specified environments. However, some studies indicate that there are still some challenges in vSLAM [12,13]. For example, pure rotation, fast motion, high computation cost, and scale problems. The studies [13,14,15] have shown that VIO, which is the integration of vSLAM and IMU, is more accurate and reliable than the other image-based methods and can solve the problems of fast motion and pure rotation. 

An IMU can estimate the attitude information of the target objects and has the advantages of low cost, lightweight, and a high data rate. Nevertheless, IMU suffers from data drifting and inaccuracy issues. Many researchers use IMU data for multi-sensor fusion for UAV navigation [16,17,18]. One of the recent popular research fields that fuse IMU and camera data is the visual–inertial navigation systems (VINS), including VIO and visual–inertial simultaneous localization and mapping (VI-SLAM) [15,19,20,21]. VINS utilizes the high IMU data rate and the camera’s rich visual information to estimate a UAV’s accurate and robust state. Two types of cameras are often used for VINS, including monocular and stereo. Monocular cameras are flexible, lightweight, and low-cost but do not provide depth information. On the other hand, a stereo camera can provide a depth map, but it is equivalent to two monocular cameras mounted side by side, which results in higher power consumption and computation cost. This study focuses on the monocular VIO navigation systems due to their efficiency and lightweight, which is more suitable for small UAVs.

The VINS can be categorized into two types, optimization-based and filtering-based. Popular algorithms for optimization-based and filtering-based are VINS-Mono [15] and robust visual inertial odometry (ROVIO) [22], respectively. Both are open-sourced and state-of-the-art monocular visual–inertial navigation algorithms. VINS-Mono is a robust and nonlinear visual–inertial state estimator that utilizes IMU preintegration, sliding window, and global optimization. ROVIO is an iterated extended Kalman filter (IEKF)-based approach where patch features are tracked by considering the IMU-predicted motion, and photometric errors are implemented as innovation terms in the IEKF update steps.

Several open-sourced monocular VIO algorithms were compared in the study [14], and VINS-Mono was the most accurate VIO algorithm among all the ones it compared. However, a UAV needs to move fully in three axes to ensure the completion of the initialization process of VINS-Mono, and the process would take up a lot of computer resources. In the real tests, VINS-Mono is updated at 9 Hz and cannot run in real-time for low-cost devices. On the other hand, the ROVIO algorithm had relatively inaccurate results and the highest frequency data rate in the study [14]. In contrast to the VINS-Mono algorithm, ROVIO was updated at 33 Hz and can run in real-time for low-cost devices in the actual test. ROVIO adopts a new idea for the feature points propagation and update of camera frames. However, it encounters some challenges. IMU is not able to obtain an accurate position and velocity. Even IMU is able to remove the bias and noise, but it still has scale factor and long-term drift problems. ROVIO performs better than the other VIO algorithms in small environments, such as the EuRoc dataset and indoor environments. However, some studies have started to test on large-scale environments, such as ADVIO datasets [23] or TUM datasets [24]. The results show that ROVIO does not perform well in ADVIO and TUM datasets, and scale and long-term drift problems obviously occur. However, due to the advantages of the high data rate and real-time capability, ROVIO is selected to be implemented in this study. An approach is proposed to reduce the scale problem and long-term drift, and scale correction is a solution.

To estimate the scale correction, some research [25,26,27,28] used range sensors such as ultrasonic, altimeter, radar, and depth camera to evaluate the metric scale of a feature point in the camera frame. However, these works often assumed that cameras horizontally look downward at the feature points, which was usually not the case since UAVs flew with angles. Stereo or multiple cameras were also popular options for VIO algorithms for the extra depth information [29,30]. However, additional sensors mean extra cost, weight, space, power, and processing time.

Using artificial landmarks to improve and correct UAV positioning is also a popular and efficient method. QR-code was used to calibrate the position of a mobile robot navigated via vSLAM in [31], and the proposed method was verified to improve the robot positioning when vSLAM is implemented. However, this method must ensure that the camera faces the artificial landmark perpendicularly to detect it. Lebedev et al. presented a technique that used the ArUco marker to adjust UAV’s position at the landing phase [32]. In [33], Xing et al. used the ArUco marker to correct the long-term drift problem of an underwater robot. Artificial landmarks are efficient and reliable assistants for calibrating and repairing robot positions. To the authors’ best knowledge, no research had tried to improve ROVIO algorithm’s long-term drift problem by an artificial landmark. The goal of this study is to develop a practical non-GPS navigation for the delivery UAVs to provide the services in urban area where GPS signal is unreliable and easy to be blocked by buildings.

In summary, the primary objective of this study is to propose a landmark-based method that can estimate the scale correction and reduce the long-term drift problem produced by the ROVIO algorithm without any external sensor. The scale estimation and correction with a landmark-based VO algorithm assistant are integrated with the output of ROVIO algorithm by using the EKF. The proposed method is proven to outperform the original ROVIO algorithm.

## 2. Scale Estimation and Correction with a Landmark Assistant

### 2.1. Flow Chart of the Proposed Approach

**Figure 1:**
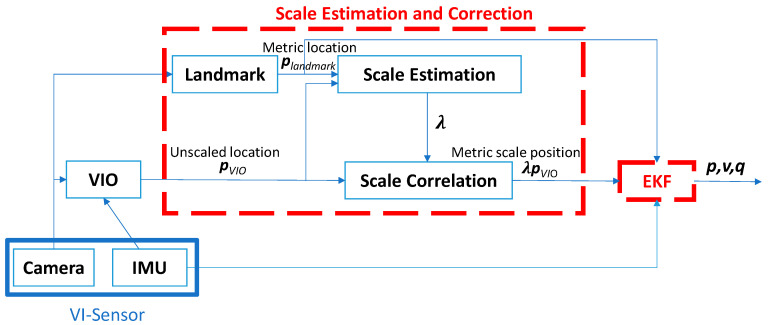
Flow chart of the proposed approach.

The methodology of this study is divided into two parts, scale estimation and the EKF integration, as shown in Figure 1. The VIO algorithm used in this research is the ROVIO algorithm. In scale estimation, a known landmark and VO algorithm are adopted to estimate the relative precision trajectory. Then, this trajectory is used to obtain the scale correction by the measurements of ROVIO and the VO algorithm with landmark detection. The difference between VIO and VO is that VIO is fused with IMU data but VO does not. After the scale correction is obtained, it is used to correct the original ROVIO, and the EKF is utilized to integrate the sensor data and the scale estimation results to predict the dynamic motion and position of the UAV. Figure 1 shows the flow chart of the overall approach. $p_{VIO}$ and $p_{landmark}$ represent the positions of the UAV estimated from the VIO algorithm and the known landmark, respectively. The vector, λ**,** is the vector of scale correction, estimated using the least-squares method to reduce trajectory errors of the ROVIO during the takeoff and landing phases of the UAV. The state vectors, ***p***, ***v***, and ***q***, are the estimated position, velocity, and quaternion vectors, respectively.

### 2.2. Coordinate Systems and Landmark Detection

**Figure 2:**
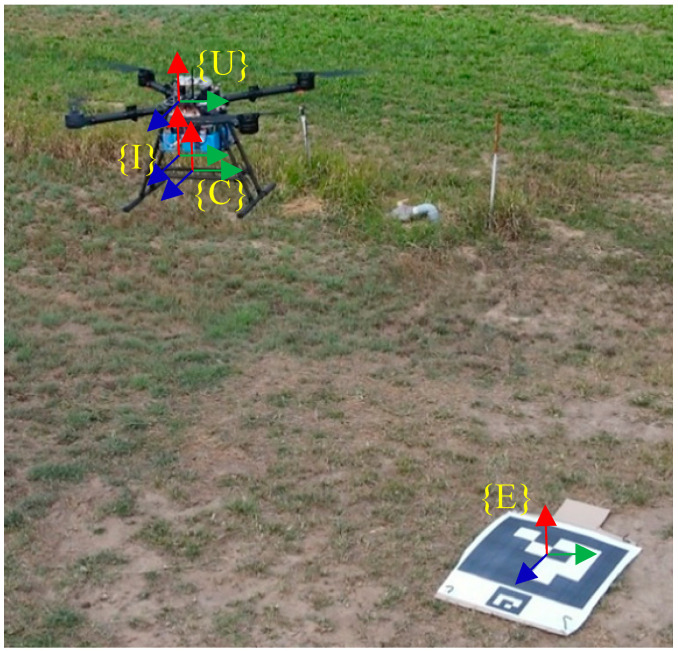
Coordinate frames of the whole system.

The application scenario of the proposed approach focuses on the UAVs operated in a GPS-denied environment and taking off and landing at the same location with an ArUco marker to improve the stability and reliability of the vision-based navigation system. The proposed method solves the scale correction and long-term drift problem of VIO during the takeoff phase and provides relatively accurate position information to UAVs, which can let UAVs return to the takeoff location. The integrated sensor fusion system is defined by four different coordinate frames, IMU sensor frame {I}, East-North-Up (ENU) frame {E}, camera frame {C}, and UAV frame {U} as shown in Figure 2. ENU frame, {E}, is the world frame in this study, and its origin is set on the ArUco marker, which is placed near the takeoff location. Camera frame, {C}, is utilized to estimate the translation and rotation between the camera and the ArUco landmark. 

**Figure 3:**
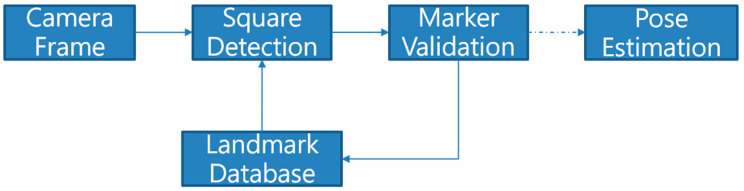
Flow chart of the landmark detection and VO estimation.

The marker detection and Perspective-n-Points (PnP) methods are utilized to accomplish the trajectory estimation. Figure 3 shows the flow chart of the landmark detection and the camera’s pose estimation based on the ArUco marker. The center of the marker is defined as the origin of the world frame, and the size and identifier of the applied landmark are defined in the database. The contour detection function of the OpenCV library is used to detect square shapes. After detecting the square contour, the cv2.findContours function will return the feature points of the detected contour on the image. Since the PnP method can determine the camera pose and location with respect to a marker, it is used to estimate the dynamic motions, which are translation and rotation, of the camera. Therefore, the $p_{landmark}$ is the position of the UAV determined by using the VO estimation of the camera with respect to the ArUco marker at the takeoff and landing location. 

### 2.3. VIO Algorithm and Scale Estimation

Both ROVIO and the proposed approach utilize the EKF to combine different data sources, but the data sources and state vectors of them are different. ROVIO tightly fuses inertial measurements and visual data with the means of an IEKF [13]. The proposed approach fuses the inertial measurements with two measurements, which are the VO outputs from the landmark and the corrected ROVIO outputs using the estimated scale correlation. When the landmark is detected, the VO algorithm is triggered and outputs the data to update the EKF. When the scale correction is determined and the landmark is not detected, the corrected ROVIO outputs are used to update the EKF.

In the scale estimation process, a known landmark is applied to estimate the accurate trajectory. However, the scale estimation process is only executed when the UAV is flying over the takeoff location and the landmark is detected by the VO algorithm. Because the landmark is placed at the takeoff location, the scale estimation process and the position update are only activated in the takeoff and landing phases. The estimated trajectory and the VIO algorithm are integrated to estimate the scale correction of the positioning results. In this study, the ROVIO is the selected VIO algorithm, which will compare the performance with the VINS algorithm. It uses the photometric error in the innovation term of the IEKF in the ROVIO algorithm, and the dynamics of the bearing vector and distance parameter are the novelty of ROVIO. It also derives a new math formula for the feature-point propagation term in the IEKF. Further detail on ROVIO can be found in the study [13].

The state of the ROVIO is defined as:

(1) $$x=\left(p\;\;v\;\;q\;\;b_{a}\;\;b_{\omega }\;\;R_{CI}\;\;T_{CI}\;\;\mu_{0}\cdots \mu_{N}\;\;\rho_{0}\cdots \rho_{N}\right),$$

where ***p*** is the position, ***v*** is the velocity, ***q*** is the orientation presented in quaternion, $b_{a}$ is the additive bias of acceleration term in IMU frame, $b_{\omega }$ is the additive bias of angular velocity term in IMU frame, $R_{CI}$ is the rotation matrix from IMU from to camera frame, $T_{CI}$ is the translation matrix from IMU from to camera frame, $\mu_{i}$ is the bearing vector in the image frame, and $\rho_{i}$ is the distance parameter of feature points in the world frame. More details of ROVIO are shown in the study [13]. The position outputs of ROVIO are the estimated trajectory of VIO, $P_{VIO}$, in Figure 1.

After estimating trajectory by landmark and VIO, the relative trajectory is used in a simple cost function as shown in Equations (2)–(4) to estimate the scale correction with the ordinary least-squares method:

(2) $$\lambda P_{\mathrm{VIO}}+b=\;P_{landmark\;},$$

(3) $$\lambda =\left[\begin{array}{ccc} \lambda_{x} & 0 & 0\\ 0 & \lambda_{y} & 0\\ 0 & 0 & \lambda_{z} \end{array}\right]$$

(4) $$minimize\;\;\;\|\lambda x-{y\|}^{2}=\sum_{𝒾=1}^{m}{\left(a_{i}x_{i}-y_{𝒾}\right)}^{2},$$

where ***x*** is ***P***_VIO_, the output trajectory from VIO, and **y** is $P_{landmark\;}$, the relative trajectory estimated by VO algorithm with landmark detection. λ is the matrix of scale estimation in 3 axes. ***b*** is the position bias vector of ***P***_VIO_. Since we set the landmark is the origin of the world frame, the b is set to zero vector in our study. After detecting the landmark and using Perspective-n-Points, the position of the camera with respect to the origin of the landmark is estimated in the world frame. When the initial point is estimated, this study uses it to define the new origin point of the camera frame and a new trajectory of VO.

### 2.4. Sensor Fusion

To accelerate the scale correction and the proposed sensor fusion process as shown in Figure 1, EKF is used to integrate the IMU, VIO with scale correction, and VO estimated by landmark. IMU is used in the time update, and the positions estimated from VIO with scale correction and VO estimated by landmark are used in the measurement update. In this study, the camera faces downward. The problem with the gimbal lock will occur. The quaternion is selected to propagate the attitude iteration instead of Euler angles to overcome the problem.

(5) $$q=q_{w}+q_{x}𝓲+q_{y}𝓳+q_{z}𝓴$$

where $q_{w},\;q_{x},\;q_{y},\;q_{z}$ are scalars, and 𝓲,𝓳,𝓴 are unit vectors. Then, the rotation matrix to convert the body frame to the world frame can be represented by the quaternion, as shown below:

(6) $$R\left(q\right)=\left[\begin{array}{ccc} \begin{array}{c} 2\left(q_{w}^{2}+q_{x}^{2}\right)-1\\ 2\left(q_{x}q_{y}+q_{w}q_{z}\right)\\ 2\left(q_{x}q_{z}-q_{w}q_{y}\right) \end{array} & \begin{array}{c} 2\left(q_{x}q_{y}-q_{w}q_{z}\right)\\ 2\left(q_{w}^{2}+q_{y}^{2}\right)-1\\ 2\left(q_{y}q_{z}+q_{w}q_{x}\right) \end{array} & \begin{array}{c} 2\left(q_{x}q_{z}+q_{w}q_{y}\right)\\ 2\left(q_{y}q_{z}-q_{w}q_{x}\right)\\ 2\left(q_{w}^{2}+q_{z}^{2}\right)-1 \end{array} \end{array}\right],$$

In this study, the Kalman filter-based method is considered instead of the optimization-based or fix-lag-based method to process sensor fusion due to the constraints of computing power and weight on the onboard computer of UAVs for real-time applications. Some fix-lag methods use the optimization method in the update phase, such as IEKF in the ROVIO algorithm, to make the system more accurate. However, IEKF requires more computing power and memory size, which will increase power consumption and reduce the endurance of the UAV operation. 

In the EKF time-update process, the information on IMU is used to propagate the states. The state vector of the EKF is as shown below:

(7) $$X=\left[\begin{array}{cccccccccc} V_{x} & V_{y} & V_{z} & P_{x} & P_{y} & P_{z} & q_{w} & q_{x} & q_{y} & q_{z} \end{array}\right]$$

where ***V*** and ***P*** denote the velocity and position in the VIO frame, and ***q*** is the quaternion in the body frame. The outputs of IMU are the acceleration and the angular velocity, denoted as a and ω, respectively. The calibration and correction of the biases for inertial sensors have been carried out in the ROVIO algorithm and sensor calibration process. In Equation (7), the position vector is corrected by using the measurements from ROVIO and landmark updates. The continuous dynamic model of the state vector can be represented as:

(8) $$\dot{V}=a$$

(9) $$\dot{P}=V$$

(10) $$\dot{q}=\frac{1}{2}q⨂\omega$$

Then, the continuous dynamic model is digitalized to a discrete form for implementation on an onboard computer.

(11) $$P_{k}^{-}=P_{k-1}+V_{k-1}∆t+\frac{1}{2}\left(R_{b}^{w}\left(a\right)+g\right)∆t^{2}$$

(12) $$V_{k}^{-}=V_{k-1}+\left(R_{b}^{w}\left(a\right)+g\right)∆t$$

(13) $$q_{k}^{-}=q_{k}⨂q\left[\omega ∆t\right]$$

where the $R_{b}^{w}$ is the direct cosine matrix from the body frame of the UAV to the world frame.

When receiving the new data from IMU, the system starts to iterate propagation steps and predict. Quaternion will be updated by integrating angular velocity to the delta angle and transferring the delta angle to quaternion derivatives. Then, the nonlinear equations are used to drive the Jacobian matrix with the system transition matrix $A_{k}$.

(14) $${\hat{x}}_{k|k-1}=f\left({\hat{x}}_{k-1|k-1},u_{k}\right)$$

(15) $$P_{k|k-1}=F_{k}P_{k-1|k-1}F_{K}^{T}+Q_{k}$$

The size of the Jacobian matrix, $F_{k}$, in this study, is 10×10 and it is defined as:

(16) $$F_{k}=\frac{\partial f}{\partial x}=\left[\begin{array}{ccc} \frac{\partial f_{1}}{\partial x_{1}} & \cdots & \frac{\partial f_{1}}{\partial x_{n}}\\ \vdots & \ddots & \vdots \\ \frac{\partial f_{n}}{\partial x_{m}} & \cdots & \frac{\partial f_{n}}{\partial x_{m}} \end{array}\right],$$

In this study, the EKF can be updated with two measurements, which are the position outputs of ROVIO and the VO algorithm. One is the VO outputs estimated by landmark. The other is the VIO with scale correction after the scale correction is estimated and the landmark is not detected by the VO algorithm. When the UAV is returning to the takeoff location, the trajectory estimated by the landmark is used again to update the scale correction. 

On the other hand, after estimating the scale correction, VIO with scale correction is applied to the measurement update process. In this study, only the position is utilized to update the scale correction without additional pose information. When receiving measurement information, the system starts to compute the measurement matrix, *H*, and the correction term.

(17) $$K=PH^{T}{\left(HPH^{T}+R\right)}^{-1}$$

(18) $$r=z_{k+1}-z_{k+1|k}$$

(19) ∆X=Kr

After estimating ∆X term, the system starts to correct the propagated state

(20) $$X=X_{k+1|k}+∆X$$

## 3. System Setup and Ground Test

### 3.1. System Setup

**Figure 4:**
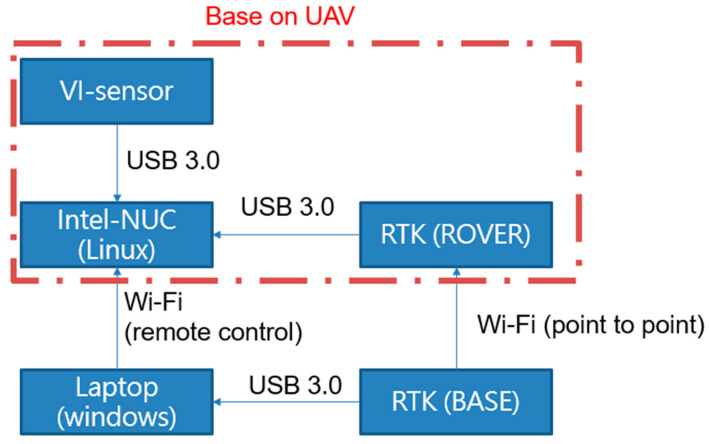
The platform of the flight test.

The block diagram of the developed VIO system for UAVs is shown in Figure 4. In the flight tests, a laptop is used to be the ground control station (GCS) for monitoring the flight tests, and the putty software and Wi-Fi are used to be the remote-control interface of the onboard computer (Intel NUC) to collect the data from different sensors, such as the VI-sensor and RTK rover receiver. In the flight tests, RTK data are not feedbacked to the flight control computer. A RTK base station is set up at GCS to transmit the RTK correction data to the rover on the UAV.

**Figure 5:**
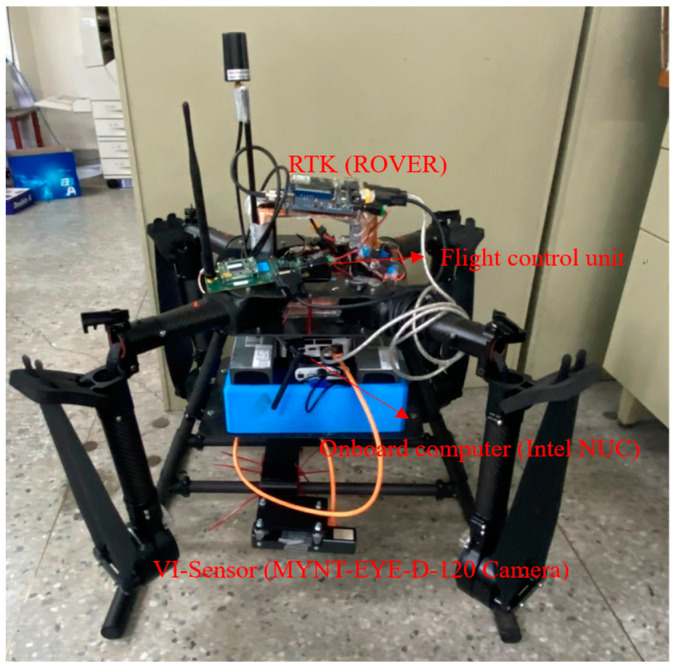
The UAV system used in the experiment.

Intel NUC is running with Linux Ubuntu 16.04, and some tools and packages, such as ROS, are installed. The MYNT-EYE-D-120 Camera is selected as the VI-sensor to collect the image and IMU data because it can provide calibrated IMU outputs, and the SDK is available with some VIO algorithms. The image resolution is 2560 × 720, and the frame rate is up to 60 FPS. The sampling rate of calibrated IMU outputs is 200 Hz. The RTK system provides the ground truth for the tests of the proposed landmark-based scale correction of VIO for UAVs. The base station equips a Hemisphere MD Eclipse P328 and the rover a Mindware MD Eclipse P327, enabling the simultaneous tracking of a lot of satellite signals, including GPS, GLONASS, and Galileo, making it the most robust and reliable solution for different applications. After the base station completes the survey, the rover will converge to centimeter level in 30~60 s. The real test results show that the 3D precision achieved an accuracy of 0.01 m, which is defined in one sigma statistical probability. The overall UAV system and its onboard equipment are shown in Figure 5.

### 3.2. Sensor Calibration

**Figure 6:**
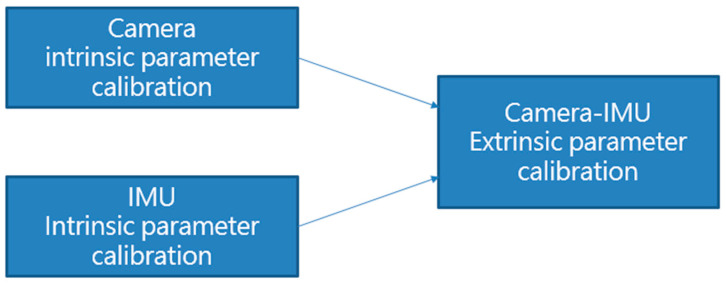
Flow chart of sensor calibration.

The reliability of ROVIO and the VO algorithm with landmark detection relies on the outputs of the camera and IMU; therefore, accurate data are essential to improve the output quality of the sensors. The proposed scale correction process adopted the calibrated IMU data and outputs of ROVIO and the VO algorithm to improve the position output of ROVIO, and the scale correction does not feedback to the states of the ROVIO algorithm. Therefore, before the flight tests, the adopted sensors, such as the camera and IMU, must be calibrated. The calibration flowchart is shown in Figure 6. The Kalibr visual–inertial calibration toolbox [34] is used to calibrate the camera intrinsic and camera–IMU extrinsic parameters. The imu_utils toolbox [35] is used to estimate IMU extrinsic parameters.

In practice, the types of cameras can be divided into a fisheye, wide angle, monocular, and stereo camera. Some cameras can produce high distortion, and the distortion can be divided into radial distortion and tangential distortion. While the distortion is zero at the optical center of the image plane, it increases with the distance away from the optical center. The correction of radial and tangential distortion parameters, denoted as $d_{r}$ and $d_{t}$, are represented below:

(21) $$d_{r}=1+k_{1}r^{2}+k_{2}r^{4}+k_{3}r^{6}$$

(22) $$d_{t}=\left[\begin{array}{c} 2p_{1}v+p_{2}\left(r^{2}+2u^{2}\right)\\ p_{1}\left(r^{2}+2v^{2}\right)+2p_{2}u \end{array}\right]$$

where $k_{1}$, $k_{2}$ and $k_{3}$ are the radial distortion coefficients, r is the Euclidean distance between the distorted image point and the optical center, $\left[u;\;v\right]$ is the distorted image point, and $p_{1}\;\mathrm{and}\;p_{2}$ are the tangential distortion coefficients. The distortion coefficients can be used to correct the acquired images. The corrected distorted image point can be determined as below:

(23) $$\left[\begin{array}{c} u_{corrected}\\ v_{corrected} \end{array}\right]=d_{r}\left[\begin{array}{c} u\\ v \end{array}\right]+d_{t}$$

For the IMU calibration, only the noise term is calibrated in this study, and the scale and misalignment of the three axes are referred to the calibrated matrix offered by the manufacturer. The IMU is kept static for more than two hours to collect the accelerations and angular rates data. Then, the collected IMU data are calibrated in the imu_utils toolbox to estimate white noise and bias instability. 

In the ROVIO algorithm, since the visual and IMU data are the main data source, precise rotation and translation relationships of the camera and IMU frames are necessary. If the errors of rotation outputs are larger than three degrees, some VIO algorithms will fail in the initial step or obtain bad results. The rotation and translation matrices are the results of the camera–IMU extrinsic parameter calibration, which is essential for data to be transferred to the same origin for the ROVIO calculation. The Kalibr toolbox is applied to complete the camera–IMU extrinsic parameter calibration. The adopted VI-sensor is faced to a desired chessboard with different poses. After receiving the data, the image and IMU data are inputted into the Kalibr toolbox to estimate the rotation and translation relationship between the camera and IMU. The results of the IMU calibration are as follows:

(24) $$R_{C}^{I}=\left[\begin{array}{ccc} 0.0175676806 & 0.9998138786 & -0.0079739995\\ -0.999843192 & 0.0175493192 & -0.0023691525\\ -0.002228773 & 0.0080143697 & 0.99996540062 \end{array}\right]$$

(25) $$t_{C}^{I}=\left[\begin{array}{c} 0.0655053034\\ -0.0163654956\\ -0.0016507275 \end{array}\right]$$

where $R_{C}^{I}$ is the rotation matrix and $t_{C}^{I}$ is the translation matrix.

### 3.3. Ground Test

**Figure 7:**
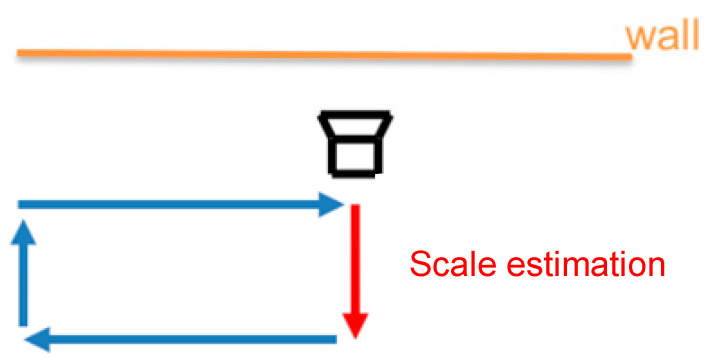
Ground test environment (square loop). The orange line denotes the wall, the red line is the scale estimation process, and the blue lines are the trajectory without the landmark assistant.

The ground test is carried out before the flight test to verify that the proposed landmark-based VIO is viable in real tests and performs better than the ROVIO and VINS-mono. The evaluation can be divided into two parts: scale correction estimation and the EKF integration, which simulates the operation of the UAV in a GPS-denied environment to take off and return to the launch location. The testing environment has a small 150 mm × 150 mm landmark attached to the wall, and the VI-sensor always looks forward at the same wall to simulate that the VI-sensor installed on the UAV looks downward when the UAV takeoffs and returns to the launch location, as shown in Figure 7. The proposed VIO and original ROVIO algorithms are compared by using root-mean-square errors (RMSE).

**Figure 8:**
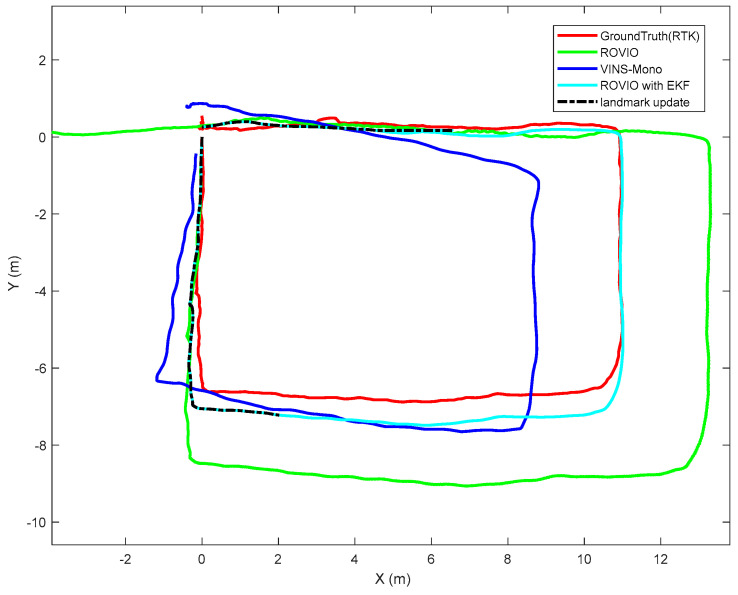
Ground test results.

**Figure 9:**
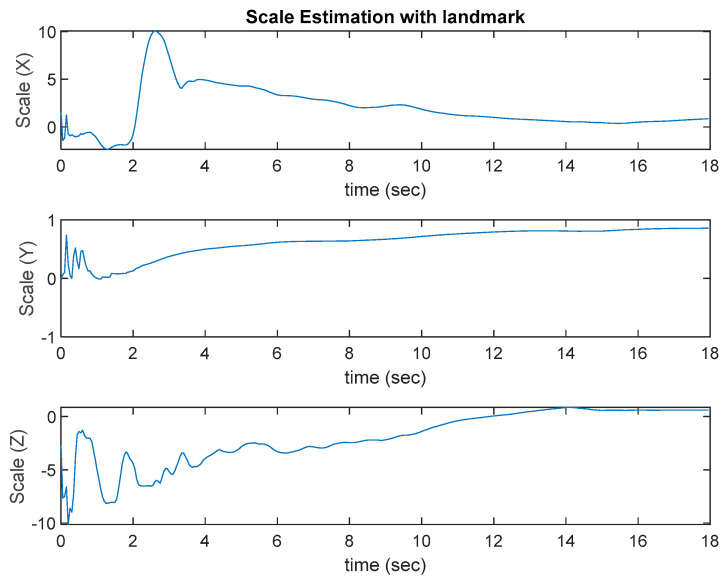
Scale estimation with a landmark.

The test platform moves slowly in a square loop. The results are shown in Figure 8. The ROVIO algorithm has scale and long-term drift problems, and the VINS-Mono algorithm has precision problem. The proposed VIO algorithm with the landmark update improves the scale and long-term drift problems. In Figure 8, ROVIO with EKF denotes the proposed method that integrates the scale correction estimation with the landmark assistant and EKF to improve the outputs of ROVIO. The scale estimation results are shown in Figure 9.

To examine how efficient the algorithm can solve the long-term drift problem, the final estimated location of the original ROVIO, ROVIO with the GPS-based scale estimation, ROVIO with the landmark-based scale estimation, and the proposed algorithm are compared with the ground truth. The results of “ROVIO with GPS”, “ROVIO with landmark”, and “ROVIO with EKF” are obtained from the fusion of EKF with different measurements, which are the positions from GPS, VO algorithm with landmark detection, and the combination of the VO algorithm with landmark detection and the corrected ROVIO outputs using the estimated scale correlation, respectively. The target location in Table 1 shows the distance between the final estimated location and the ground truth. The results show that the final estimated location of the proposed algorithm, ROVIO with landmark assistant to estimate the scale correction, is the closest to the ground truth among all the tested algorithms. To evaluate the performance of the EKF update process with different measurements, the RMSE results of original ROVIO, ROVIO with GPS, ROVIO with landmark, the proposed scale correction algorithms, and the ground truth are calculated and shown in Table 1. The location estimated by the proposed approach has fewer scale problems and is much more accurate than the other approaches.

## 4. Flight Test Results and Discussion

**Figure 10:**
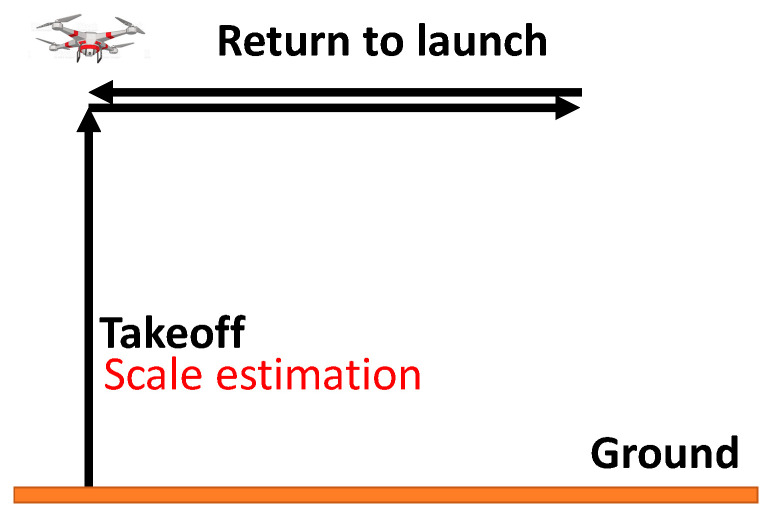
Design of the flight test route. The orange line denotes the ground, and the black lines are the flight trajectory.

In the flight test, the landmark was placed on the ground near the UAV takeoff location, which is represented as (0,0). A waypoint was sent to the UAV from the ground control station. The UAV is designed to take off, fly to the waypoint, and return to launch, as shown in Figure 10. The scale estimation process happened when the UAV took off with the VI-sensor looking downward and facing the landmark. Two flight cases were conducted. In the scale estimation process, Case 1 demonstrated that the VI-sensor moved slowly and fully in three axes, and Case 2 showed that the VI-sensor moved fast and did not fully move in three axes to conduct different scale estimation conditions. 

**Figure 11:**
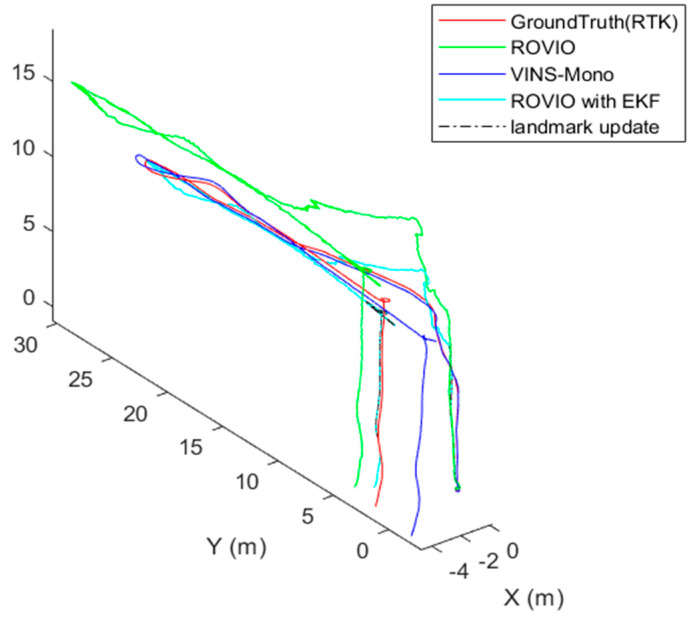
The flight test results of Case 1.

**Figure 12:**
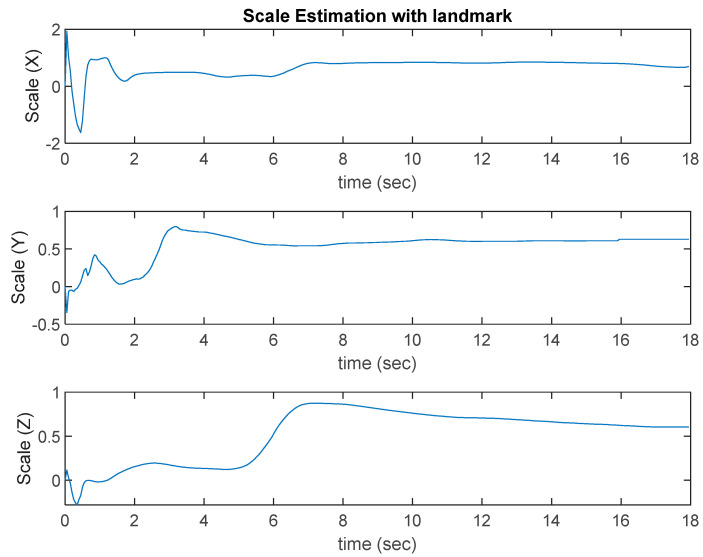
Scale estimation with the landmark in Case 1.

In Case 1, as Figure 11 shows, the VINS-Mono algorithm performs well. The ROVIO algorithm has an obvious scale problem and long-term drift. Moreover, when the UAV moves quickly, the estimated location of the ROVIO algorithm performs the worst, and the overshoot occurs when the UAV takes off. As shown in Figure 11**,** the proposed VIO algorithm with a landmark update provided an accurate location in the return-to-launch process and performed better than VINS-Mono. Figure 12 shows the results of the scale estimation with a landmark in Case 1.

**Figure 13:**
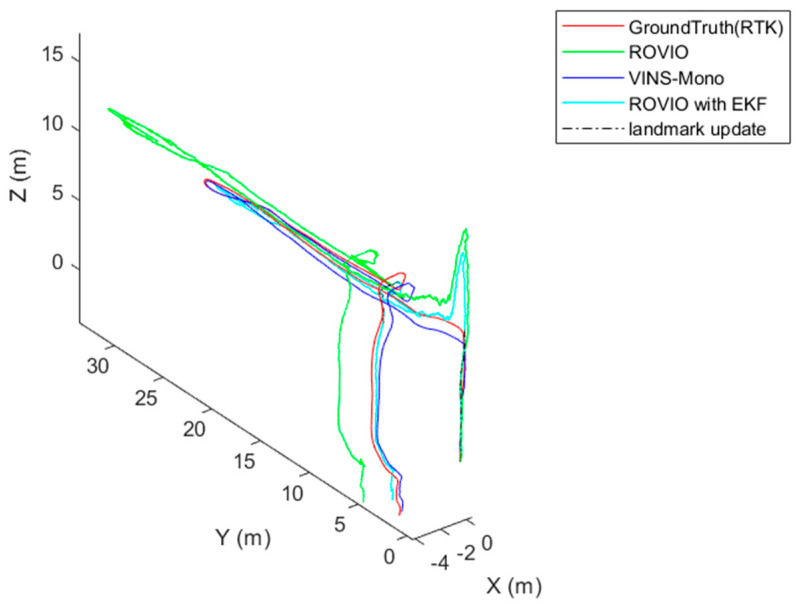
Flight test results of Case 2.

**Figure 14:**
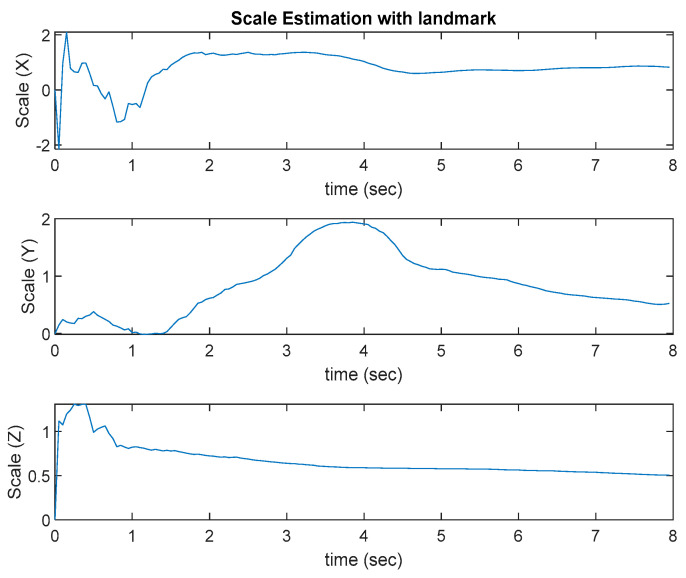
Scale estimation with the landmark in Case 2.

In Case 2, VINS-Mono also has good performance, and ROVIO still has the scale, long-term drift, and overshoot problems, as shown in Figure 13. With scale correction, the proposed algorithm estimates the location closer to the ground truth, but the peak problem is not solved effectively. Figure 13 also shows that even though the VI-sensor can only detect the landmark in a few frames when the UAV returns to launch, it still helps to improve the accuracy of the estimated target location. Figure 14 shows the results of scale estimation with the landmark in Case 1.

Similar to the ground test, Table 2 shows the distance errors between the final estimated location and ground truth, and Table 3 shows the RMSE of the performance comparison between different algorithms and the ground truth. 

The results show that the proposed VIO can estimate scale successfully and reduce long-term drift, especially when the landmark information is captured. However, there is a peak problem. The scale can be better and effectively corrected by moving the VI-sensor fully on the three axes.

## 5. Conclusions

In this study, a landmark-assistant VIO algorithm is presented to be used in a real-world GPS-denied environment. The proposed VIO algorithm provides a complete scale correction estimation and uses the landmark to reduce the long-term drift problem. The proposed method is used to process the dataset collected from the ground and flight tests and is compared with the ROVIO and VINS-Mono algorithms. In the scale correction estimation process, the VI-sensor needs to be moved fully on three axes to successfully estimate the scale correction. In experiments, VIO with scale correction can obtain a better performance, and the result is close to the ground truth. However, it cannot ideally reduce the long-term drift problem and the momentary external force.

With the EKF sensor fusion, the rate of output positioning points is increased. With the landmark update in the measurement update, the VIO algorithm can effectively reduce the long-term drift, and the target location is closer to the ground truth. Some of the future considerations to improve the performance are shown:

1. Add more experiment designs to complete full movements in three axes.
2. Add external force estimation in the algorithm.
3. Use a GPS timestamp to synchronize the time of the camera and IMU.
4. Add external pose information in the measurement update process.

## Figures and Tables

**Table 1:** Ground test results.

	ROVIO	ROVIO with GPS	ROVIO with Landmark	ROVIO with EKF
Target Location	4.2167 m	3.9842 m	3.7498 m	0.037 m
RMSE	3.7469 m	1.9756 m	2.1976 m	0.3432 m

**Table 2:** Flight test results: final estimated location compares with the ground truth.

	ROVIO	ROVIO with GPS	ROVIO with Landmark	ROVIO with EKF
Case 1	1.6687 m	1.4254 m	1.4596 m	1.116 m
Case 2	7.1529 m	6.1271 m	5.9645 m	2.4478 m

**Table 3:** Flight test results: RMSE of the performance comarision between different algorithms and the ground truth.

	ROVIO	ROVIO with GPS	ROVIO with Landmark	ROVIO with EKF
Case 1	3.2097 m	2.2386 m	1.8626 m	1.7431 m
Case 2	8.4588 m	6.0043 m	5.7631 m	5.1649 m

## Data Availability

Not applicable.
